# Draft Genome Sequence of Extremely Drug-Resistant *Pseudomonas aeruginosa* (ST357) Strain CMC_VB_PA_B22862 Isolated from a Community-Acquired Bloodstream Infection

**DOI:** 10.1128/genomeA.01092-16

**Published:** 2016-10-20

**Authors:** Agila Kumari Pragasam, Francis Yesurajan, George Priya Doss C, Biju George, Naveen Kumar Devanga Ragupathi, Kamini Walia, Balaji Veeraraghavan

**Affiliations:** a Christian Medical College, Vellore, Tamil Nadu, India; b Vellore Institute of Technology, Vellore, Tamil Nadu, India; c Indian Council of Medical Research, New Delhi, India

## Abstract

Extremely drug-resistant *Pseudomonas aeruginosa* strains causing severe infections have become a serious concern across the world. Here, we report draft genome sequence of *P. aeruginosa* with an extremely drug-resistant profile isolated from a patient with community-acquired bloodstream infection in India.

## GENOME ANNOUNCEMENT

*Pseudomonas aeruginosa* is an important causative agent of several infections, especially in critically ill and immunocompromised patients. It is well known for its unique ability to resist most of the antipseudomonal agents by both chromosomal and acquired resistance (1). Carbapenem-based combinations are the drugs of choice for treating infections due to multidrug-resistant (MDR) *P. aeruginosa* (2). However, the emergence of resistance to these agents limits their use in clinical practice.

In this report, we present the draft genome sequence of *P. aeruginosa* (CMC_VB_PA_B22862), isolated from a community-acquired bloodstream infection at Christian Medical College, South India. Antimicrobial susceptibility testing of antipseudomonal agents found resistance to ceftazidime, cefotaxime, piperacillin-tazobactam, imipenem, meropenem, aztreonam, levofloxacin, amikacin, netilmicin, and gentamicin, except colistin. The MICs were 32, 32, and 16 µg/ml for imipenem, meropenem, and doripenem, respectively. Since CMC_VB_PA_B22862 was resistant to all the agents except colistin (extremely drug resistant), whole-genome sequencing was done to look for the molecular mechanism of antimicrobial resistance.

Whole-genome sequencing (WGS) was performed using Ion Torrent PGM platform using 200-bp read chemistry. Sequencing was performed as per the protocol recommended by Life Technologies. Raw reads were assembled *de novo* using the assembler SPAdes software version 4.4.0.1 in Torrent suite server version 4.4.3. Sequencing generated 6,774,328 bp of sequence with 199 contigs (≥500 bp) of 36× coverage. The genome was annotated using Pathosystems Resource Integration Centre (Patric) database (https://www.patricbrc.org) (3) and Rapid Annotation using Subsystem Technology (RAST; http://rast.nmpdr.org/) (4). Upon annotation, 8,195 coding sequences (CDSs) with 29 and 45 antimicrobial resistance genes were found as per ARDB and CARD, respectively. In addition, 209 and 124 virulence factors have been found as per the VFDB and Victors databases, respectively. Similarly, several insertional sequences, including IS*pa32*, IS*pa39*, and IS*rs014*, belonging to the IS*3* family, and a transposon, Tn*3* (IS*pa38*), were identified by ISFinder analysis (5). In addition, CRISPRFinder analysis revealed four confirmed and possibly three additional clustered regularly interspaced short palindromic repeat (CRISPR) sequences (6).

The sequence type was found to be ST357 (https://cge.cbs.dtu.dk/services/MLST/) based on the pubMLST database. Further analysis of antimicrobial resistance genes in Res Finder found different classes of resistance genes. This includes beta-lactams (*bla*_OXA-10_, *bla*_OXA-50_, *bla*_VEB-1_, and *bla*_PAO_), aminoglycosides [*aadA1* and *aph(3)-llb*], fosfomycin (*fosA*), sulfonamide (*sul1*), tetracycline [*tet*(A)], chloramphenicol (*catB7*), and trimethoprim (*drfB2*). However, no carbapenemase genes were found by ResFinder 2.1 (https://cge.cbs.dtu.dk//services/ResFinder/). In contrast, Patric identified specialty genes, including hypothetical proteins belonging to a metallo-beta-lactamase superfamily. Further, chromosomal gene mutations in the genes coding for MexAB, MecCD, MexEF, and MexXY efflux pumps were noted. Also, one mutation each was found in *gyrA* and *parE*, and two mutations in *parC* conferring resistance to fluoroquinolones were identified. Altogether, plasmid-mediated genes were found to be present against various classes of antimicrobials. However, mutations in chromosomal genes were also found to be a significant contributing factor for antimicrobial resistance.

### Accession number(s).

The WGS of CMC_VB_PA_B22862 was deposited in DDBJ/EMBL/GenBank under the accession number LRXM00000000. The version described in this article is LRXM01000000.
